# Prediction of *E. coli* Concentrations in Agricultural Pond Waters: Application and Comparison of Machine Learning Algorithms

**DOI:** 10.3389/frai.2021.768650

**Published:** 2022-01-11

**Authors:** Matthew D. Stocker, Yakov A. Pachepsky, Robert L. Hill

**Affiliations:** ^1^Environmental Microbial and Food Safety Laboratory, United States Department of Agriculture–Agricultural Research Service, Beltsville, MD, United States; ^2^Oak Ridge Institute for Science and Education, Oak Ridge, TN, United States; ^3^Department of Environmental Science and Technology, University of Maryland, College Park, MD, United States

**Keywords:** machine learning, microbial water quality, *E. coli*, irrigation water, food safety

## Abstract

The microbial quality of irrigation water is an important issue as the use of contaminated waters has been linked to several foodborne outbreaks. To expedite microbial water quality determinations, many researchers estimate concentrations of the microbial contamination indicator *Escherichia coli (E. coli)* from the concentrations of physiochemical water quality parameters. However, these relationships are often non-linear and exhibit changes above or below certain threshold values. Machine learning (ML) algorithms have been shown to make accurate predictions in datasets with complex relationships. The purpose of this work was to evaluate several ML models for the prediction of *E. coli* in agricultural pond waters. Two ponds in Maryland were monitored from 2016 to 2018 during the irrigation season. *E. coli* concentrations along with 12 other water quality parameters were measured in water samples. The resulting datasets were used to predict *E. coli* using stochastic gradient boosting (SGB) machines, random forest (RF), support vector machines (SVM), and k-nearest neighbor (kNN) algorithms. The RF model provided the lowest RMSE value for predicted *E. coli* concentrations in both ponds in individual years and over consecutive years in almost all cases. For individual years, the RMSE of the predicted *E. coli* concentrations (log_10_ CFU 100 ml^−1^) ranged from 0.244 to 0.346 and 0.304 to 0.418 for Pond 1 and 2, respectively. For the 3-year datasets, these values were 0.334 and 0.381 for Pond 1 and 2, respectively. In most cases there was no significant difference (*P* > 0.05) between the RMSE of RF and other ML models when these RMSE were treated as statistics derived from 10-fold cross-validation performed with five repeats. Important *E. coli* predictors were turbidity, dissolved organic matter content, specific conductance, chlorophyll concentration, and temperature. Model predictive performance did not significantly differ when 5 predictors were used vs. 8 or 12, indicating that more tedious and costly measurements provide no substantial improvement in the predictive accuracy of the evaluated algorithms.

## Introduction

Food safety is a fundamental public health concern which is threatened when waters with poor microbial quality are used for the irrigation of fresh produce. In the U.S. and around the world, regulatory or advisory thresholds on the microbial quality of irrigation waters are based on the concentrations of *Escherichia coli* (*E. coli*) measured in the water source (US Food and Drug Administration., 2015; Allende et al., 2018; Wen et al., 2020). Irrigation with water of substandard microbial quality has been implicated with foodborne outbreaks associated with the consumption of contaminated produce (Nygård et al., 2008; Kozak et al., 2013; Gelting et al., 2015). Additionally, it is known that pathogenic microorganisms transferred with irrigation water can internalize into crop tissues which extends their persistence and reduces the efficacy of post-harvest washing (Solomon et al., 2002; Martinez et al., 2015). Ensuring that water used for irrigation meets the recommended criteria is vital for protecting public health and reducing incidences of foodborne outbreaks.

Concentrations of fecal indicator organisms, primarily *E. coli*, are commonly used to characterize microbial water quality. Researchers have investigated the relationships between fecal microorganisms and water quality parameters such as dissolved oxygen, pH, turbidity, and nutrient levels with a goal of improving timeliness and predictability of microorganism concentrations in a water source (Francy et al., 2013; McEgan et al., 2013; Stocker et al., 2019). Such dependencies have varied considerably across studies. This may at least partially be explained by the complexity and non-linearity of relationships of fecal microorganisms with multiple water quality parameters, which in turn exhibit complex relationships.

Machine learning (ML) algorithms have been extensively shown to outperform traditional multivariate analyses in numerous aquatic ecology studies where the two analyses have been compared (Quetglas et al., 2011). The advantage of machine-learning methods is in their ability to mimic linear relationships between dependent and independent variables. Machine learning models are also able to assess associations and quantify predictability in the absence of the knowledge needed for developing process-based models (Thomas et al., 2018). Within the field of microbial water quality, several researchers have used ML algorithms to develop models which could be used to make rapid water quality determinations in rivers, streams, Great Lakes beaches, groundwater, drinking water wells, and water distribution systems (Brooks et al., 2016; Mohammed et al., 2018, 2021; Panidhapu et al., 2020; Tousi et al., 2021; Weller et al., 2021; White et al., 2021).

A large number of ML algorithms have been proposed and implemented in a variety of different research disciplines (Kuhn and Johnson, 2013). Common research goals are (a) to obtain an accurate predictive relationship between the predictors and target variables, and (b) to determine and rank the most influential predictors. By achieving these goals, researchers may be able to eliminate unimportant predictors from measurement programs which can potentially save a great deal of time and resources. The application of ML regressions in the field of microbial water quality is relatively new (Park et al., 2015; García-Alba et al., 2019; Stocker et al., 2019; Abimbola et al., 2020; Ballesté et al., 2020; Li et al., 2020; Belias et al., 2021; Wang et al., 2021). For agricultural waters, research into predicting *E. coli* concentrations using ML was done for streams (Weller et al., 2021), but so far no studies have utilized ML regressions to predict *E. coli* concentrations in agricultural irrigation ponds which serve as an important source of irrigation water across the United States and abroad.

The objectives of this work were (i) evaluate and compare the capabilities of several popular ML algorithms for predicting concentrations of *E. coli* from water quality parameters in irrigation ponds, and (ii) to determine the most influential predictors for the estimation of *E. coli* concentrations using a multiyear dataset.

## Methods

### Field Sites and Data Collection

Two working irrigation ponds in Maryland were sampled during the 2016–2018 growing seasons. Sampling typically occurred on a biweekly schedule between May and August. Specific details of the sampling procedures can be found in Pachepsky et al. (2018) and Stocker et al. (2021). Briefly, each pond was sampled in a grid-like pattern the maps of which are shown in Supplementary Figure 1. Pond P1 is located in central Maryland and provided irrigation water to the surrounding fruit fields. The northern part of the pond is surrounded by a forested area whereas the other two sides are covered by grasses or bushes. The fields around P1 received chemical fertilizers prior to planting each year in late March or early April.

Pond P2 is an excavated pond located on the University of Maryland's Wye Research and Education Center (WREC) which is located on Maryland's Eastern Shore. The pond receives water from a culvert at the northern end (location 12 on the map). The pond is surrounded by dense brush vegetation along the perimeter as well as by several trees planted further up from the banks. To the northern end of the pond is a small riparian area that surrounds an ephemeral creek while the southernmost portion has a wetland area that leads into the Wye River.

Along with each water sample that was collected, a YSI sonde was used to determine characteristics of the water quality in that sampling location. In 2016, a YSI MPS 556 (Yellow Springs Instruments, Yellow Springs Ohio) unit was used to measure dissolved oxygen (DO), pH (pH), specific conductance (SPC), and temperature (C) which were measured *in-situ*. At the laboratory, a Lamotte turbidimeter was used to measured turbidity (NTU) of the samples. In 2017, a YSI EXO 2 was used to measure all of the previously described water quality variables as well as the concentrations of chlorophyll (CHL), phycocyanin (PC), and fluorescent dissolved organic matter (*f* DOM). In 2018, the same YSI EXO 2 sonde was used but additional laboratory measurements were performed. These included ammonium (${\text{NH}}_{4}^{+}$), orthophosphate (${\text{PO}}_{4}^{3-}$), total nitrogen (TN), and total carbon (TC). Ammonium was measured using an ion-selective probe (CleanGrow, United Kingdom) which was calibrated prior to analysis which occurred on the same day as sample collected. Orthophosphate was run on a SEAL AQ300 discrete nutrient analyzer (SEAL Analytical, Mequon, Wisconsin). Total carbon and TN were analyzed on a Vario TOC cube (Elementar Hanau, Germany) using high temperature combustion and tandem TN_b_ and TC detectors.

*E. coli* enumeration was performed based on EPA method 1603 (US Environmental Protection Agency., 2005) which utilizes membrane filtration. Briefly, 100 ml of pond water was vacuum filtered through 0.45 μm membrane filters. Filters were then placed onto modified mTEC agar (BD Difco, Sparks, MD) and incubated for 2 h at 37°C and then 22 h at 44.5°C. After incubation, colonies that were purple in color were counted as *E. coli*. Each sample was duplicate plated and the resulting counts were then averaged.

### Machine Learning Algorithms and Implementation

Several ML algorithms as well as a multiple linear regression (MLR) were compared in this work. The stochastic gradient boosting algorithm (SBG) builds the prediction model from an ensemble of weak models which in this case are decision trees (Friedman, 2002). Models are built in a step-wise fashion where at each step a weak model is fitted to a subsample of the training data drawn at random without replacement. The term “gradient boosting” comes from the fact the model is trying to minimize a loss function by tweaking parameters until a minimum value is reached. The R package “gbm” (Greenwell et al., 2020) was used develop SGB models. Parameters for the SGB algorithm are the number of trees (n.trees), the number of splits in the trees (interaction.depth), the learning rate (shrinkage), and minimum number of observations in terminal nodes of trees (n.minobsinnode).

The k-nearest neighbors (kNN) algorithm implements a non-parametric approach which computes distances from test datasets to the neighboring training datasets and uses these distances to determine the predicted value for the test dataset. The distances are computed from predictor values for training and test datasets. The number of neighbors (*k*) used for the prediction is the single parameter for this algorithm. A Euclidean distance measure was used to determine nearest neighbors. The “kknn” package (Schliep et al., 2016) was used to develop kNN models.

The support vector machines (SVM) algorithm finds the global minimum in the predictor (Cristianini and Shawe-Taylor, 2000). Support vector machines automatically select their model size and prevent overfitting by using special form of the regression cost function that balances accuracy and flexibility (Vapnik et al., 1995). Support vector machines neglects small errors which makes it robust and computationally treatable. It employs mapping to use linear regression while the relationship between original (not mapped) predictor and output variables is non-linear. The “kernlab” package (Karatzoglou et al., 2019) was used to run the SVM algorithm with a radial basis function kernel which has two control parameters. The γ parameter defines how far the influence of a single training example reaches and the parameter C controls the overfitting prevention.

The random forest (RF) algorithm creates predictions by generating many decision trees and combining their predictions in a weighted average giving the final prediction. The RF algorithm also has a built-in mechanism for preventing overfitting by random selection of inputs for the individual trees. As implemented in the ranger package (Wright and Ziegler, 2017), the algorithm includes three control parameters. The mtry parameter controls overfitting by determining the number of variables to randomly select at each split in the trees. The min.node.size parameter sets the minimum number of observations in a terminal node. The number of trees was kept at 500 to reduce computational intensity and because out-of-bag error did not appreciably change after this number of trees.

One of the outcomes of running RF algorithms is determining the most influential features that effect the model output. A random-forest based recursive feature elimination (RF-RFE) algorithm was applied to each dataset to determine the most influential predictors. The result of this procedure is to (i) find the subset of predictors with the minimum possible generalization error and (ii) to select the smallest possible subset of predictors which provide the optimal accuracy in model performance (Granitto et al., 2006). Within the algorithm, at each iteration feature importance is calculated based on the overall effect on the residual error and then the least important predictors are removed. The recursion is needed to address the problem that for some measures of relative importance, the results can change substantially over different subsets of the entire predictor list.

All ML algorithms as well as a MLR model were applied within the “caret” (classification and regression training) R package (Kuhn, 2008). This package contains functions that streamline training for complex ML regression and classification problems. The package facilitates the optimization and execution of ML algorithms and uses other R packages as functions for creating models. For each of the above-listed ML algorithms, we used the “caret” package to perform repeated cross-validation when fitting the ML models to the datasets. A default 10-fold cross-validation was performed with five repeats and then the results were averaged. The “caret” package was also applied to perform the recursive feature elimination. Algorithm tuning was performed during cross-validation and tuning was performed to minimize the average root mean squared error $\bar{RMSE}.$ The “caret” package contains a grid search function for control parameter tuning which was utilized in this study. The optimal control parameters as well as most influential variables were identified during cross-validation.

### Comparison of Algorithm Performance Metrics

The average root mean square error $\bar{RMSE}$, coefficient of determination $\bar{R^{2}}$, and mean absolute error $\bar{MAE}$ were the metrics used to evaluate algorithm performance in this study. Averaging of *RMSE*, *R*^2^ and *MAE* was done across 50 values of those statistics obtained for all cross-validation folds and repeats. The probability of the averages being the same for a pair of algorithms was determined from the corrected Student statistics *t*_*c*_:


(1) $$\begin{array}{r} t_{c,\;RMSE}=\frac{{\bar{RMSE}}_{1}-{\bar{RMSE}}_{2}}{\sqrt{h\sigma_{RMSE_{1}-RMSE_{2}}^{2}}};t_{c,\;MAE}=\frac{{\bar{MAE}}_{1}-{\bar{MAE}}_{2}}{\sqrt{h\sigma_{MAE_{1}-MAE_{2}}^{2}}}; \end{array}$$

$$\begin{array}{r} t_{c,\;R^{2}}=\frac{\bar{R_{1}^{2}}-\bar{R_{2}^{2}}}{\sqrt{h\sigma_{R_{1}^{2}-R_{2}^{2}}^{2}}}; \end{array}$$

where subscripts “1” and “2” refer to the first and the second compared algorithms, $\sigma_{RMSE_{1}-RMSE_{2}}^{2}$, $\sigma_{MAE_{1}-MAE_{2}}^{2}$, and $\sigma_{R_{1}^{2}-R_{2}^{2}}^{2}$ are variances of differences between the values of *RMSE, MAE*, and *R*^2^, respectively, obtained for the same fold and repeat, *h* is the variance correction term proposed by Bouckaert and Frank (2004) to account for the fact that that values of the metrics in the 50 individual random samples are not independent as they are obtained by the random subsampling of the same dataset. The value of *h* is determined as


(2) $$\begin{array}{r} h=\frac{1}{k\cdot r}+\;\frac{n_{testing}}{n_{training}} \end{array}$$

where *k* is the number of folds and *r* is the number of repeats, *n*_*training*_ instances are used for training, and the remaining *n*_*testing*_ instances for testing in each of runs. The *t*_*c*_ statistics in (1) have the Student *t* distribution with *k*·*r* – 1 degrees of freedom. The value of the ratio *n*_*testing*_/*n*_*training*_ was 0.1 in this work as recommended by Bouckaert and Frank (2004). Having the value and knowing the distribution of *t*_*c*_, one can estimate the probability of the differences between the average metrics from two models being equal to zero. Bouckaert and Frank (2004) referred to this test statistic as the “corrected repeated k-fold cv test.”

Normalized root-mean-square-error (*NRMSE*) and mean absolute error (*NMAE*) were also computed by dividing the $\bar{RMSE}$ and the $\bar{MAE}$, respectively, by the range of *E. coli* concentrations (e.g., maximum–minimum concentration) and then multiplying by 100 for each year and predictor set. *NRMSE* and *NMAE* show the percentage of algorithm error relative to the spread of data.

### Data Preprocessing and Analysis

*Escherichia coli* count data was log-transformed prior to statistical analysis. All observations of 0 CFU 100 ml^−1^ were assigned a value of 0.5 to facilitate the log-transformation (US Environmental Protection Agency., 2005). Rows with missing values were removed prior to analysis. Data was not normalized or standardized before analysis. Preliminary findings showed that these operations did not substantially affect algorithms performance and in many cases resulted in poorer predictions.

To examine model performance and variable importance using different combinations of predictors, we created three different predictor sets. These included set A which is DO, pH, SPC, NTU, and C, set AB which is set A plus *f* DOM, PC, and CHL, and set ABC which is set AB plus ${\text{PO}}_{4}^{3-}$, ${\text{NH}}_{4}^{+}$, TN, and TC. Models with the Set A were developed for the individual years 2016 to 2018 and the combined 3-year dataset. Models with Set AB predictors were developed for 2017 and 2018, and models with the set ABC predictor were built only for 2018 where all 12 of the parameters were measured.

A separate study was performed to evaluate the models developed with the combined P1 and P2 datasets as opposed to models developed with separate P1 and P2 datasets. Only the predictor set A was evaluated in this exercise because these predictors were present in all years of observations and across ponds. The combined dataset was modeled with and without the introduction of a categorical variable “site” that labeled the data from different ponds.

## Results

### Summary of Monitoring Data

The P1 2016A, 2017A, and 2018A datasets contained 50, 126, and 138 samples, respectively, after row removal due to missing values. The P1 2017AB and 2018 AB were 126 and 138 samples, respectively, while the 2018ABC dataset was 92 samples after row removal. For P2, the sample set sizes were 97, 148, and 202 samples for 2016A, 2017A, and 2018A, respectively and the 2017AB and 2018AB had the same dimensions as the A scenario set. The P2 2018ABC dataset had 202 samples.

*Escherichia coli* and other water quality variable concentration averages and standard errors are shown in Supplementary Table 1. The two ponds contained similar concentration ranges of *E. coli* in general although the P1 2017 dataset year contained consistently higher concentrations. The 2016 datasets for each pond contained higher amounts of missing values of *E. coli* concentrations compared to the 2017 and 2018 datasets which had relatively few (<5%).

Values of most of the water quality parameters were similar between ponds with a few exceptions. Pond 2 in 2017 and 2018 had elevated DO concentrations compared to other instances. CHL and PC were also several times higher in P2 than in P1 in 2017 and 2018. The 2016 NTU concentrations at P2 were considerably higher than in other cases. Orthophosphate concentrations were about 16.5 times greater at P2 than in P1 in 2018. However, P1 had an average ${\text{NH}}_{4}^{+}$ concentration which was about three times greater than in P2. Average SPC values varied between 142.59 and 166.95 across the two ponds. Average *f* DOM values were higher in P2 in 2017 and 2018 than P1 for each corresponding year.

### Evaluation of Machine Learning Algorithm Performance

The $\bar{RMSE}$ values and standard errors of RMSE are presented in Table 1. As expected, the MLR performed substantially worse than the ML algorithms for both ponds and for all years and predictor sets. The differences between the performance of the ML algorithms were less substantial. Overall, the differences among average SVM, RF, and SGB $\bar{RMSE}$ for the same ponds and years were <10%. The kNN demonstrated relatively larger spread of differences between its RMSE and $\bar{RMSE}$ of other ML algorithms and the range of those differences was from 1.5 to 24.9%.

**Table 1:** Average root-mean-squared errors (RMSE) of logarithms of *E. coli* concentrations predicted with four machine learning algorithms and multiple linear regression.

**ML Algorithm**	**Predictor set A**				**Predictor set AB**			**Predictor set ABC**
	**2016**	**2017**	**2018**	**2016–2018**	**2017**	**2018**	**2017–2018**	**2018**
**Pond P1**								
SGB	**0.247** **±** **0.011**	**0.250** **±** **0.012**	0.354 ± 0.015	0.343 ± 0.009	0.257 ± 0.012	0.348 ± 0.012	0.325 ± 0.008	0.336 ± 0.011
kNN	0.279 ± 0.016	0.276 ± 0.012	0.395 ± 0.015	0.366 ± 0.010	0.283 ± 0.016	0.385 ± 0.016	0.356 ± 0.011	0.361 ± 0.016
MLR	0.452 ± 0.033	0.287 ± 0.013	0.556 ± 0.016	0.504 ± 0.009	0.288 ± 0.014	0.518 ± 0.014	0.461 ± 0.008	0.447 ± 0.012
RF	0.255 ± 0.015	**0.250** **±** **0.012**	**0.346** **±** **0.015**	**0.334** **±** **0.010**	**0.244** **±** **0.013**	**0.338** **±** **0.013**	**0.322** **±** **0.010**	**0.334** **±** **0.014**
SVM	0.269 ± 0.013	0.255 ± 0.012	0.384 ± 0.013	0.356 ± 0.009	0.260 ± 0.012	0.382 ± 0.014	0.344 ± 0.009	0.371 ± 0.014
**Pond P2**								
SGB	0.332 ± 0.011	0.422 ± 0.013	0.381 ± 0.007	0.402 ± 0.007	0.428 ± 0.015	0.375 ± 0.008	0.403 ± 0.007	0.314 ± 0.009
kNN	0.370 ± 0.015	0.416 ± 0.015	0.405 ± 0.008	0.423 ± 0.008	0.424 ± 0.012	0.401 ± 0.009	0.408 ± 0.009	0.396 ± 0.009
MLR	0.421 ± 0.016	0.463 ± 0.012	0.434 ± 0.008	0.506 ± 0.008	0.467 ± 0.012	0.418 ± 0.009	0.506 ± 0.006	0.391 ± 0.010
RF	0.306 ± 0.012	**0.416** **±** **0.014**	**0.344** **±** **0.009**	**0.381** **±** **0.007**	**0.418** **±** **0.014**	**0.343** **±** **0.008**	**0.385** **±** **0.007**	**0.304** **±** **0.008**
SVM	**0.288** **±** **0.012**	0.424 ± 0.014	0.365 ± 0.008	0.404 ± 0.007	0.431 ± 0.013	0.378 ± 0.011	0.406 ± 0.009	0.340 ± 0.010

*The ± separates the average from the standard error of the mean. The smallest RMSE are shown in bold. Machine learning algorithms: SGB, stochastic gradient boosting machines; kNN, k-nearest neighbor; MLR, multiple linear regression; RF, random forest; SVM, support vector machines. Predictor sets: A—temperature (C), DO, pH, turbidity, and SPC; AB—all from A and PC, CHL, and fDOM; ABC—all from AB and ${\text{NH}}_{4}^{+}$, ${\text{PO}}_{4}^{3-}$, TN, and TC*.

#### Random Forest as the Best-Performing Algorithm

**Figure 1:**
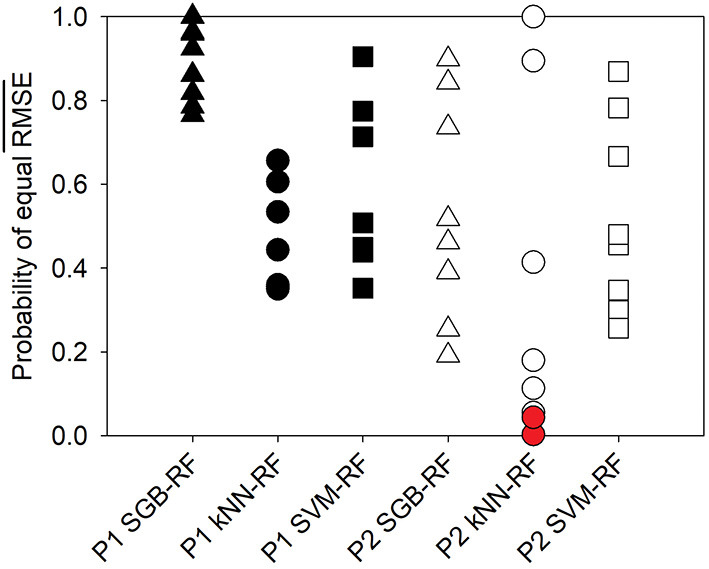
Probabilities for the mean RMSE value to be the same in the RF and other ML applications based on the corrected t-statistic. Symbols in red show statistical significance.

The RF algorithm provided the smallest $\bar{RMSE}$ value in 88% of cases. Only in 2016, the SGB and SVM algorithms, on average, provided lower RMSE values for P1 and P2, respectively. The SGB algorithm provided the second smallest $\bar{RMSE}$ in 75% of cases. Probabilities of $\bar{RMSE}$ being equal for RF and other algorithms are shown in Figure 1. The probability ranges differed between the ponds and among algorithms. Whereas the probabilities of equal $\bar{RMSE}$ for SGB and RF were high for Pond 1, Pond 2 had a greater spread of probabilities that were generally lower. Similarly, the range of probabilities of equality of $\bar{RMSE}$ for RF and kNN was much wider for Pond 2 compared with Pond 1. Probabilities of equal $\bar{RMSE}$ for RF and SVM were relatively high in both ponds. Ranges of those probabilities were similar in both ponds, unlike with other algorithms. The two significant differences in model performance occurred between the RF and kNN algorithms with the 2018 A (*P* < 0.043) and the 2018 ABC (*P* < 0.001) P2 datasets.

#### Interannual Differences in Algorithm Performance

Probabilities of the absence of differences in $\bar{RMSE}$ for pairs of years varied by year, algorithm, and pond (Supplementary Table 2). Comparisons between 2016 and 2017 performance were not significant in the P1 dataset. For P2, the SVM, RF, and SGB algorithms performed significantly better on the 2017 than the 2016 set. The 2017 predictor A set showed significantly better performance than the 2018 A set in P1 but was not found to significantly differ for any model in P2. Between the 2016 and 2018 predictor A sets, the SGB, kNN, and SVM algorithms performed significantly better for the 2016 dataset than for the 2018 dataset whereas the performance, while better for RF and MLR in 2016, did not significantly differ. The MLR model was the only model at P2 which showed significantly better performance in 2016 when compared to 2018.

#### Multiannual Algorithm Performance

When ML algorithms were applied to the three-year dataset from P1 with the predictor set A, the $\bar{RMSE}$ appeared to lie between the maximum and minimum $\bar{RMSE}$ obtained for individual years for the same pond and predictor set. For P2 and predictor set A, the $\bar{RMSE}$ of the three-year dataset were slightly higher than the largest of the individual year $\bar{RMSE}$. A similar pattern was observed with the predictor set AB and the 2-year dataset. The $\bar{RMSE}$ for P1 was between the $\bar{RMSE}$s of individual years, whereas with P2 data, the $\bar{RMSE}$ of individual years were smaller than the $\bar{RMSE}$ of the 2-year dataset.

#### Effect of the Predictor Set Expansion

**Figure 2:**
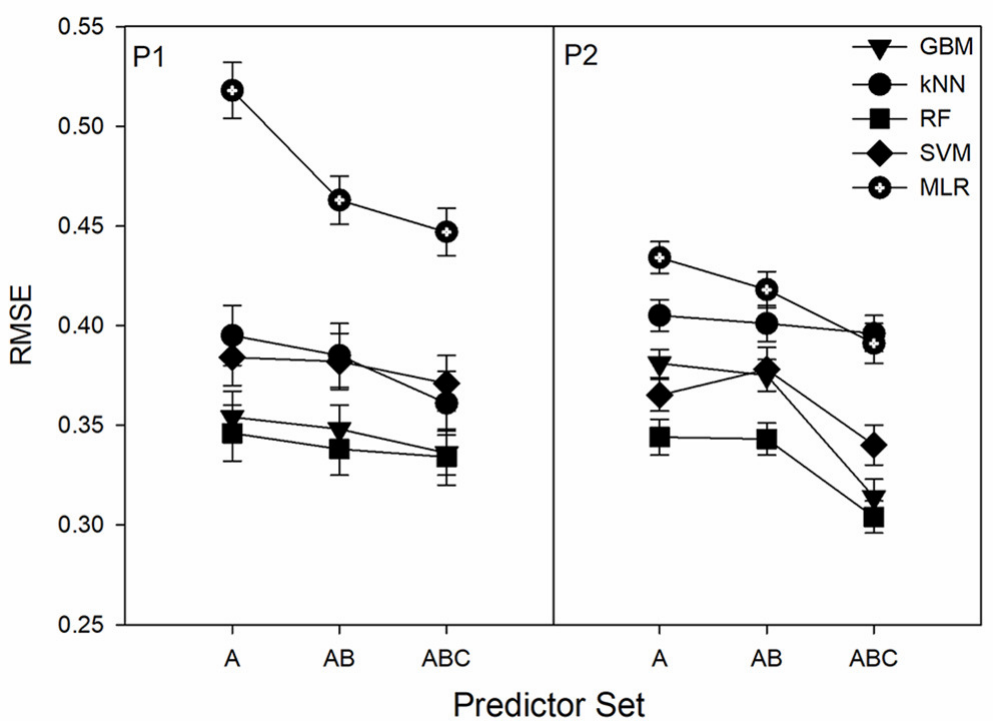
Dependence of the root-mean-squared error (RMSE) on the predictor set size. Predictor sets have 5, 8, and 12 predictors for A, AB, and ABC, respectively. Displayed results are from the 2018 datasets.

Expanding the predictor set size from A to AB in the 2017 resulted in a slight increase of $\bar{RMSE}$ in most cases with the one exception being for the RF model in P1. Transition from A to AB and then to ABC predictor sets generally led to the decrease of $\bar{RMSE}$ (Figure 2). However, in some cases there was effectively no difference (i.e., RF in P1 between AB and ABC and kNN between all sets in P2). In P1, there was a gradual decrease in RMSE with increased number of predictors whereas for P2 the largest *RMSE* decreases were between AB and ABC with kNN being the exception. In all cases the 12-predictor ABC set showed the best performance for all ML algorithms at both ponds.

#### Other Metrics of Algorithm Performance

The differences between algorithm performance measured with mean absolute error (MAE) and the determination coefficient (*R*^2^) were similar to the differences found with RMSE values (Supplementary Tables 3, 4). However, in a few instances the model with the best performance changed when analyzing different metrics. For example, the SGB model provided the best performance in the 2018 ABC predictor set in P1 and the kNN provided the best result for the 2016–2018 predictor set A for P2 when using MAE as a metric. These two were predicted most accurately by the RF model when RMSE is used. Similarly, the SGB model in P1 2017–2018 AB predictor set in P1 provided the highest *R*^2^-value and the kNN model was best in the 2017 A predictor set in P2 whereas both were predicted best by the RF model when using RMSE.

The RF algorithm provided either the best (12 cases) or the second best (4 cases) value of the average MAE. The SGB and SVM algorithms were the closest to the RF (Supplementary Table 3). Similar to the results comparing RMSE, the 2016 and 2017A and AB sets were predicted with the lowest errors according to average MAE and errors were generally lower with P1 than P2. The only significant differences in ML model performances using the MAE metric was between the RF and kNN in the P2 2018 ABC dataset (*P* = 0.004) (Supplementary Figure 2). Based on *R*^2^-values, the RF model was preferred in 12 of 16 cases with it being second in two cases and tied with the SGB model in two cases (Supplementary Table 4). Interestingly, *R*^2^-values for the 2017 A and AB sets were the lowest across models despite this year having the smallest average RMSE and MAE values. Similar to results with RMSE and MAE, kNN was the only ML model to perform significantly worse (*P* = 0.003) than the RF model which occurred for the 2017–2018 AB dataset (Supplementary Figure 3).

Normalized RMSE and MAE were calculated for all years and predictor sets for both ponds (Supplementary Tables 5, 6). The preferred algorithm did not change from those presented in Table 1 and Supplementary Table 2 for values of RMSE and MAE, since for a given dataset, RMSE and MAE values from all algorithm were divided by the same value. The percentages of the errors as shown by examination of NRMSE values (Supplementary Table 5) for the ML algorithms were generally around 10 % of the data range for the P1 2016A, 2017A, 2016–2018A, and 2017AB datasets. The P1 2018A, AB, and ABC datasets contained higher errors which were between 10 and 20%. The MLR algorithm provided consistently higher errors which were typically in the range of 15–20% except for the 2017 datasets which had <10% error. P2 contained greater relative error (15–20%) than the P1 datasets with the exception being the 2018A, AB, and ABC data in which errors were in the same range between ponds. Values of NMAE were consistently lower than those of NRMSE and were in the range of 5.2% to (RF P1 2017A) to 15.5% (MLR P2 2018 A).

#### Algorithm Performance for the Combined Pond 1 and Pond 2 Datasets

Results of combining the P1 and P2 datasets are shown in Supplementary Table 6. The SVM algorithm performed the best in terms of values of RMSE, *R*^2^, and MAE in the combined 2016 dataset whereas the RF algorithm showed the best performance in the 2017, 2018, and 2016–2018 datasets. The algorithm performance for the combined P1 and P2 dataset was never better than the performance of both ponds run individually for certain years but was typically higher than one pond and lower for the other (Table 1; Supplementary Tables 3–5). Including “site” as a categorical input generally improved the performance of the combined P1/P2 datasets across years (Supplementary Table 7).

### Recursive Feature Elimination

In almost all scenarios the RF model was shown to provide the best $\bar{RMSE}$ values when compared to the other models. For this reason, a recursive feature elimination in “caret” was performed using the RF model to determine and rank the most important features for each pond and by year. Supplementary Figure 4 shows the reduction in RMSE values for the RF models for each year. Examination of the graph shows that in most cases the models do not greatly benefit from more than five predictors but the specific predictors varied by pond and year. The predictor importance order for each year from the RF-RFE was recorded and each variable received a numeric value corresponding to the overall ranking (Table 2). In this way, the variables with the lowest scores corresponded to being picked as consistently most important.

**Table 2:** The top five important variable as determined by the recursive feature selection algorithm in caret with Random Forests.

		**Pond 1**					**Pond 2**			
**Variable set A**		**Variable set AB**		**Variable set ABC**		**Variable set A**		**Variable set AB**		**Variable set ABC**	
**Variable**	**Average rank**	**Variable**	**Average rank**	**Variable**	**Rank**	**Variable**	**Average rank**	**Variable**	**Average rank**	**Variable**	**Rank**
SPC	1.3	SPC	2.0	*f*DOM	1	C	2.0	SPC	1.5	*f*DOM	1
C	1.7	C	2.5	SPC	2	SPC	2.7	C	2.0	SPC	2
DO	3.7	*f*DOM	3.5	CHL	3	pH	3.3	pH	2.5	CHL	3
pH	4.0	CHL	4.5	TN	4	NTU	3.3	NTU	5.0	TN	4
NTU	4.3	NTU	5.5	TC	5	DO	3.7	*f*DOM	5.5	TC	5

*Variable set A was measured in 2016, 2017, and 2018. Variable set AB was measured in 2017 and 2018 and variable set ABC was measured in 2018 only. A = C, pH, NTU, SPC, AB = A + CHL, PC, fDOM. ABC = AB + ${\text{NH}}_{4}^{+}$, ${\text{PO}}_{4}^{3-}$, TN, and TC*.

Within the various predictor set scenarios, there were some parameters that were consistently highly ranked as important features. These included SPC, C, *f* DOM, CHL, and NTU. All other parameters showed largely inconsistent ranking. While SPC was consistently ranked in the top two variables for all scenarios, temperature was as well for variable sets A and AB but was ranked poorly in the ABC datasets. The PC-value was consistently ranked low in the datasets that contained this parameter. Similarly, ${\text{NH}}_{4}^{+}$ and ${\text{PO}}_{4}^{3-}$, the only ionic nutrients measured in the study were also ranked low in the ABC set. Conversely, TN and TC were ranked fourth and fifth important in both ponds in the 2018 datasets. Interestingly, the 2018 ABC parameter sets had identical rankings of the five top important features and overall similar rankings of the remaining seven parameters.

## Discussion

While model performance generally did not significantly differ, the RF model was found to provide consistently better performance than any of the other models evaluated. Several publications focusing on ML model evaluation specifically for microbial water quality purposes have reached similar conclusions (Avila et al., 2018; Chen et al., 2020; Weller et al., 2021). The SGB model was found to provide the second-best performance across all three metrics from all datasets. In several empirical ML comparison studies, it has been stated that SGB models usually outperform RF models (Maclin and Opitz, 1997; Caruana and Niculescu-Mizil, 2006; Hastie et al., 2009) but other studies report the opposite (Bauer and Kohavi, 1999; Manchanda et al., 2007; Khoshgoftaar et al., 2010). Therefore, the choice of “the best” model may be dataset-dependent which to some degree was evident in the results of this work (Table 1; Supplementary Tables 3, 4). Also, the performance of both models is dependent on how they are tuned. SGB are considered harder to tune than RF models, contain greater sensitivity of tuning parameters with regard to the output, and have a greater number of tuning parameters (Freeman et al., 2016). Both RF and SGB algorithms are tree-based but the SGB algorithm incorporates a boosting procedure whereby model fitting is additive and each new tree is fit to the residuals of the previous tree with the goal of minimizing the most egregious errors according to a specific loss function such as MSE. Because SGBs are additive, they are more susceptible to over- or underfitting which the RF model is robust to because the individual trees are independent and are averaged to create the forest. Lastly, when bagging (RF) and boosting (SGB) type algorithms have been compared, bagging has been described to provide better results when datasets are noisy or there are class imbalances (Maclin and Opitz, 1997; Khoshgoftaar et al., 2010).

The performance of the SVM and kNN algorithms was generally poorer than the RF and SGB algorithms (Table 1) but in most cases the performance did not significantly differ (Figure 1). One possibility is that both SVM and kNN algorithms have been reported to not handle missing values, near-zero variance predictors, or noisy data as well as RF or SGB algorithms (Kuhn and Johnson, 2013). The SVM algorithm typically provided lower RMSE than the kNN algorithm and this may be because SVM is robust to outliers especially when non-linear kernels are used. The RBF kernel was used in this study because it provided substantially better results than the linear or polynomial kernels (data not shown) which was also reported in the work by Weller et al. (2021) who used ML algorithms for the prediction of *E. coli* in NY streams. Several other water quality studies have also reported better performance of SVM than kNN when the two have been compared (Modaresi and Araghinejad, 2014; Danades et al., 2016; Babbar and Babbar, 2017; Prakash et al., 2018; Chen et al., 2020). Finally, the kNN has been reported to not perform well with high dimensional or highly scattered datasets which is why centering and scaling is recommended. However, in our work, this pre-processing procedure did not affect results but may explain why SVM is preferred in other water quality datasets.

Different performance metrics in general agreed with each other, but in some cases contradicted. For example, while all algorithms showed the best $\bar{RMSE}$ on the 2017 P1 dataset (Table 1), this year was ranked the worst predicted by the *R*^2^ metric (Supplementary Table 4). This is caused by the distribution of observed and predicted data along a 1:1 line. This example highlights the importance on the choice of performance metric reported in algorithm evaluation studies and the advantage of using multiple performance metrics.

We compared only five different algorithms in this study which were chosen due their popularity, but many more ML algorithms and their modifications exist and can be tested for regression-type application (Kuhn, 2008; Kuhn and Johnson, 2013; Weller et al., 2021). We chose not to run artificial neural networks (ANN) due to constraints of the dataset dimensions but other researchers have found success in applying ANN algorithms in the field of microbial water quality (Motamarri and Boccelli, 2012; Buyrukoglu et al., 2021). Other promising algorithms for water quality determinations include those founded in Bayesian statistical methods such as Naïve Bayes or Bayesian Belief Networks (Avila et al., 2018; Panidhapu et al., 2020) and the use of ensemble or model stacking methods (Buyrukoglu et al., 2021).

Several predictor variables emerged as consistently important for both ponds and across years of observations. These included *f* DOM, SPC, C, and CHL, and NTU. Positive relationships between dissolved organic matter (*f* DOM) and concentrations of planktonic fecal bacteria in water have been reported (Rincon and Pulgarin, 2004; Bouteleux et al., 2005). The relationship is likely governed by the presence of suspended organic substances which may promote the growth and survival of *E. coli* in the present study by providing nutrients, an attachment surface, and decreasing direct cellular photo-inactivation (Rincon and Pulgarin, 2004; Garcia-Armisen and Servais, 2009; Maraccini et al., 2016; KatarŽyte et al., 2018).

In both ponds and in every year NTU was positively correlated with *E. coli* concentrations (data not shown). Positive associations of fecal bacteria and NTU have been previously presented (Francy et al., 2013; Partyka et al., 2018; Weller et al., 2020) and can also be related to the level of suspended particulates which have been shown to enhance *E. coli* survival in water (KatarŽyte et al., 2018). Additionally, elevated NTU levels may indicate recent disruption of bottom sediments either by bioturbation or runoff-related mixing which results in the resuspension of fecal bacteria contained in sediments (Cho et al., 2010; Stocker et al., 2016).

The presence of CHL in the lists of most important predictors apparently reflects mutualistic relationships between algae and *E. coli* have been reported and attributed to solar shielding as well algae providing a source of labile organic nutrients which promote bacterial persistence (Englebert et al., 2008; Vogeleer et al., 2014). On average in both ponds, when chlorophyll-a (CHL-a) levels were higher, *E. coli* concentrations were lower (Supplementary Table 1). There may exist a threshold level at which there is a mutualistic relationship between *E. coli* and algae and above this level there exists competition (Ansa et al., 2011).

The concentrations of SPC were determined as most important in the largest number of cases in the study. The concentrations of SPC in water are proportional to the ion concentrations present. Ionic nutrient concentrations in water have often shown positive relationships with the concentrations of *E. coli* present (Lim and Flint, 1989; Ozkanca, 1993; Shelton et al., 2014). Recent research has also demonstrated that *E. coli* survival rates in freshwater increase with conductivity by way of reducing osmotic stress and improving membrane stability but can be detrimental above certain levels (DeVilbiss et al., 2021). Runoff reaching waterways may either have a dilutional effect and lower water conductance or increase it (Baker et al., 2019). Rapid changes in SPC may be used as an indicator of when influent such as runoff or precipitation has reached water sources and thus may provide good indication of when *E. coli* concentrations can be expected to change within a waterbody.

Temperature effects on *E. coli* persistence in the environment are perhaps the most well-documented of any other variables in the literature but may also be the most inconsistent. Numerous review and meta-analysis articles indicate *E. coli* persistence is negatively influenced by higher temperatures (Blaustein et al., 2013; Stocker et al., 2014; Cho et al., 2016). However, others have reported positive relationships (Truchado et al., 2018) while some have reported inconsistent direction of the relationship when multiple sites were included in the same study (Francy et al., 2013; McEgan et al., 2013). These diverse dependencies reveal the complexity of the relationships between *E. coli* and the predictors which govern the aquatic habitat and affect survival. Ultimately, ML algorithms are expected to handle the complex interactions and non-linear relationships better than traditional regression models in aquatic studies (Quetglas et al., 2011; Weller et al., 2021).

Through additional scenario testing (Table 2; Supplementary Tables 3, 4) it was discovered that model performances did not substantially change when the 2017 and 2018 datasets were held at a lower number of predictors. This indicates while parameters introduced in later years of the study were found to be at times more important, the core 5 predictors utilized in 2016A, 2017A, and 2018A predictor sets (C, SPC, NTU, DO, and pH) were found to be largely suitable for predicting *E. coli* concentrations in agricultural pond waters. This finding is of special interest as each additional predictor introduces additional burden on water quality characterization program. Additionally, results of this study indicate that *E. coli* concentrations in irrigation ponds may be “now casted” by using relatively cheap deployable on-line sensor suites that are used for continuous monitoring. It must be acknowledged that this study utilized measurements of a total of 12 predictors. Many additional predictors exist (e.g., ORP, total suspended solids, or various nutrient concentrations such as nitrate or ammonia) which may further improve the predictive performance of the ML algorithms or lead to the creation of similar simple and effective sets of variables as those identified in this study.

We realize that the effect of redundancy of predictors was not fully elucidated in this work. There exist multiple suggestions on redundancy removal as a preprocessing step of regressions using correlations between predictors, variance inflation estimation, or principal component analysis as a basis for the removal of covarying predictors. Multiple methods are suggested in the literature to reduce the effects of the input reduction on variable importance determinations (Bøvelstad et al., 2007). These methods tend to increase the reliability of the regression results (more data per coefficient), but at the same time, they may change the perception of the relative importance of input variables (Ransom et al., 2019). Applying these methods to several ML algorithms and assessing results presents an interesting research avenue. In this work, we limited the study by analyzing correlations between inputs. As expected, the only strong correlations were found between DO and pH (data not shown). The most likely mechanism for the observed co-linearity is photosynthetic activity in the ponds which consumes dissolved CO_2_ (raising pH) and releases DO (raising DO). We cannot exclude the effect of this correlation on the occurrence and position of DO and pH in lists of important inputs.

The algorithm performance for the combined P1 and P2 dataset was never better than the performance of both ponds run individually for certain years but was typically higher than one pond and lower for the other (Table 1; Supplementary Tables 3–5). Explanations for this may be site-specific responses of *E. coli* concentrations to differences in predictor levels in each pond which may in some cases be similar and in others dissimilar. For example, P2 had elevated levels of the photosynthetic pigments PC and CHL compared to P1. Similarly, DO and pH levels in P2 were typically higher in P2 than in P1 (Supplementary Table 1). It is therefore possible that there were different extents to the effects of these predictor levels on *E. coli* concentrations which were unique for each pond. If monitoring datasets are available for multiple water bodies, one can pool these datasets together and compare the performance between site-specific models and those developed using pooled datasets across locations. Alternatively, one may use “site” as categorical variable input which may preserve site-specific interactions between *E. coli* and predictors within a larger model. Indeed, in the present study, adding “site” (e.g., Pond 1 or Pond 2) generally improved the performance of all algorithms on the P1/P2 combined datasets (Supplementary Tables 5, 6).

The results of this work were gathered by studying only two irrigation ponds both in the state of Maryland and as such the scope of inference is limited. However, in the literature there is a lack of information regarding *E. coli* and water quality dynamics in irrigation sources let alone those involving ML algorithms. The current study suggests a framework for using ML algorithms for irrigation water quality determinations.

## Conclusions

Overall, all ML algorithms performed well in predicting *E. coli* in the datasets. The RF algorithm predicted better in more cases than the other models when assessed in terms of average values of root-mean-squared-error, coefficient of determination, and mean absolute error (MAE). However, when those performance metrics were treated as statistics, there was no significant difference between the ML algorithm performance in most cases. The MLR model consistently provided the worst results which demonstrated the non-linearity of the relationships between *E. coli* and its predictors. The recursive feature elimination exercise revealed similarities in important features across years and sites. Namely, SPC, NTU, C, CHL, and *f* DOM were found to be the most influential variables for the prediction of *E. coli* in the studied ponds. However, it was also shown that the algorithm performances were not substantially improved when predictor sets were expanded to 8 and 12 variables from the core 5 variable list (pH, DO, SPC, C, NTU). The performance of the RF model as well as its relatively simple set up and deployment indicate it may be a valuable tool for water quality managers and researchers to utilize when predicting the microbial quality of irrigation waters.

## Data Availability Statement

The raw data supporting the conclusions of this article are part of MS's doctoral dissertation which will be published in Spring of 2022. After this time the data will be made available by email request to the corresponding author, without undue reservation.

## Author Contributions

MS and YP designed the monitoring program, planned and performed the data analysis, and co-wrote the manuscript. MS oversaw and participated in the data collection. RH critically evaluated the manuscript and advised MS as needed. YP obtained funding for the project. All authors contributed to the article and approved the submitted version.

## Funding

This work was supported through the USDA's Agricultural Research Service project number 8042-12630-011-00D. RH salary was supported, in part, by the USDA National Institute of Food and Agriculture, Hatch project 1014496.

## Conflict of Interest

The authors declare that the research was conducted in the absence of any commercial or financial relationships that could be construed as a potential conflict of interest.

## Publisher's Note

All claims expressed in this article are solely those of the authors and do not necessarily represent those of their affiliated organizations, or those of the publisher, the editors and the reviewers. Any product that may be evaluated in this article, or claim that may be made by its manufacturer, is not guaranteed or endorsed by the publisher.
