# Supplementary material for: Prediction of E. coli Concentrations in Agricultural Pond Waters: Application and Comparison of Machine Learning Algorithms
Source: Front Artif Intell. 2022 Jan 11;4:768650. doi: 10.3389/frai.2021.768650 (PMC8787305; doi:10.3389/frai.2021.768650)
Supplement: Supplementary file 1 [file Data_Sheet_1.docx]

**Supplementary Material**

# Supplementary Figures


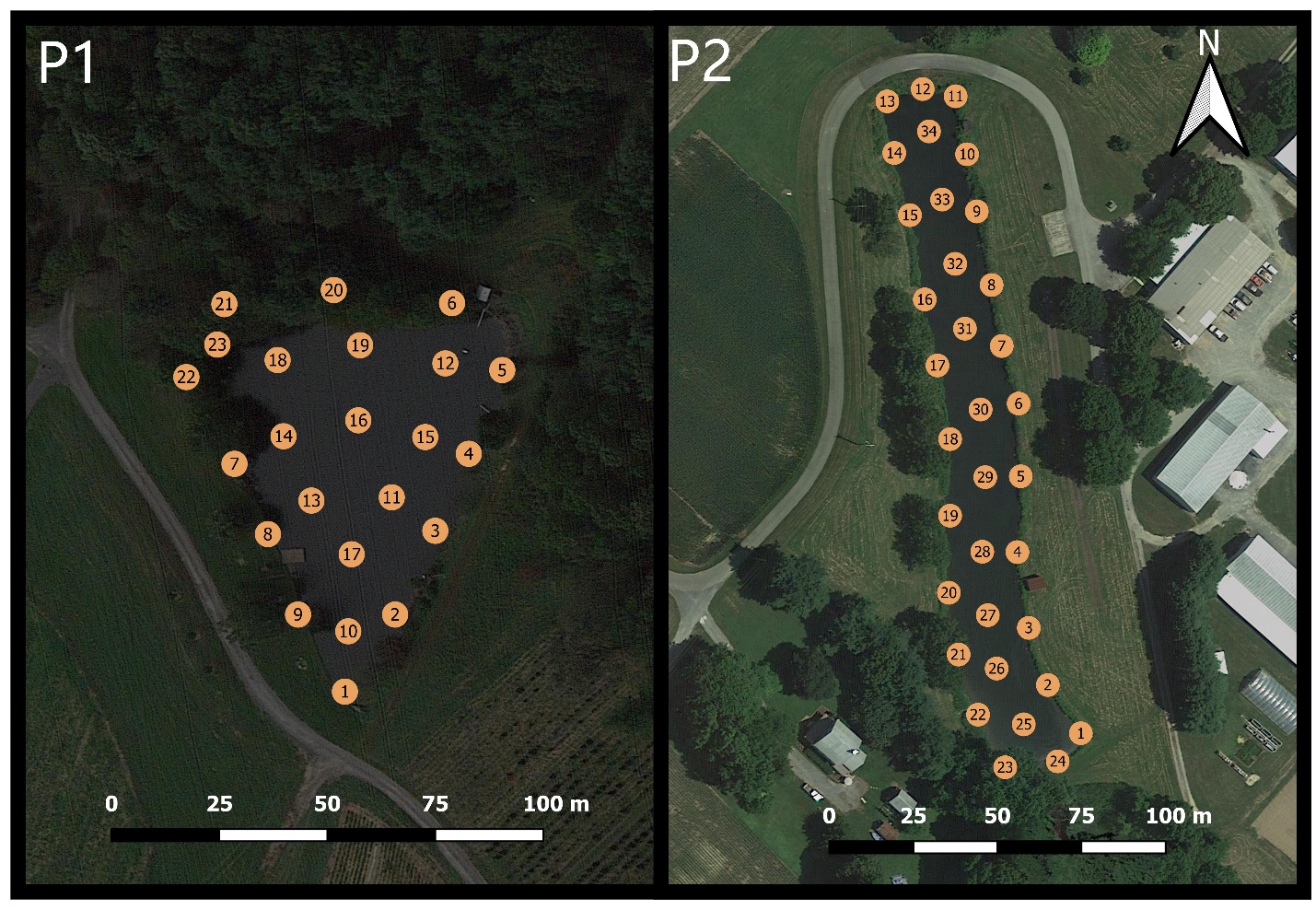


Supplementary Figure 1. Site maps for the two studied ponds. P1 had 23 sampling locations and is pictured on the left. P2 had 34 sampling locations and is pictured on the right


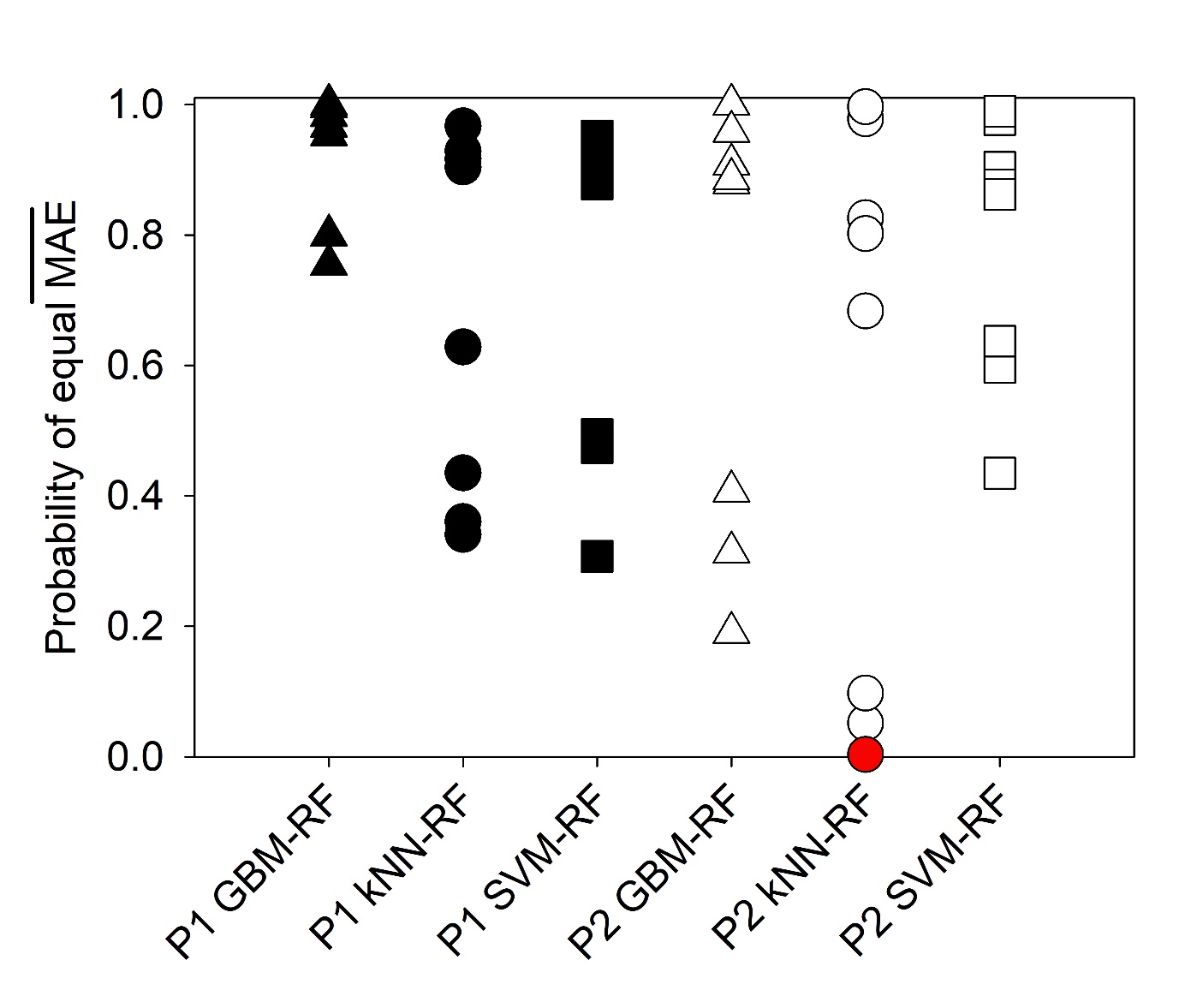


**Supplementary Figure 2**. Probabilities of MAE between the RF and other ML models. Red symbols indicate statistically significant differences.


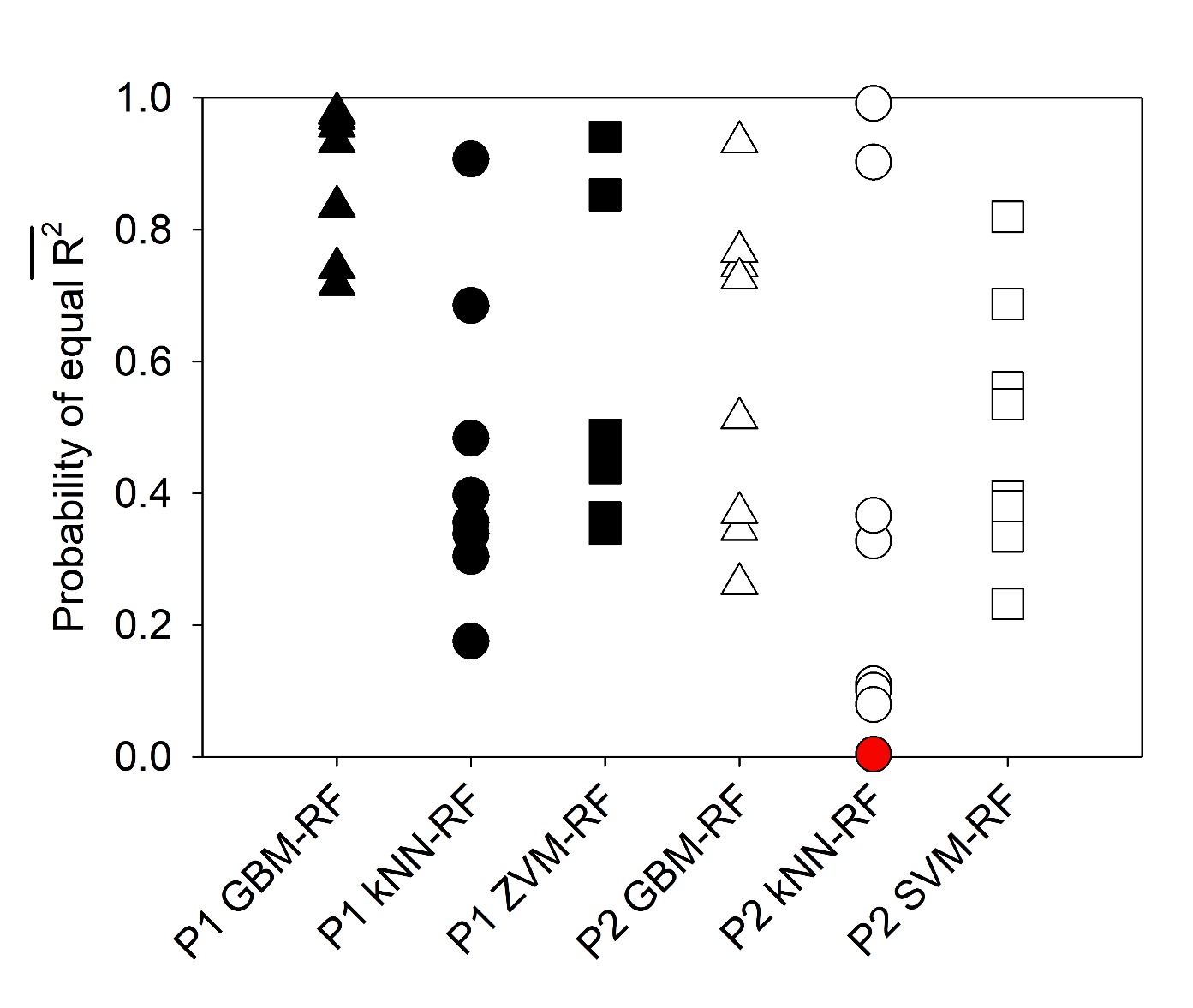


**Supplementary Figure 3**. Probabilities of R^2^ between the RF and other ML models. Red symbols indicate statistically significant differences.


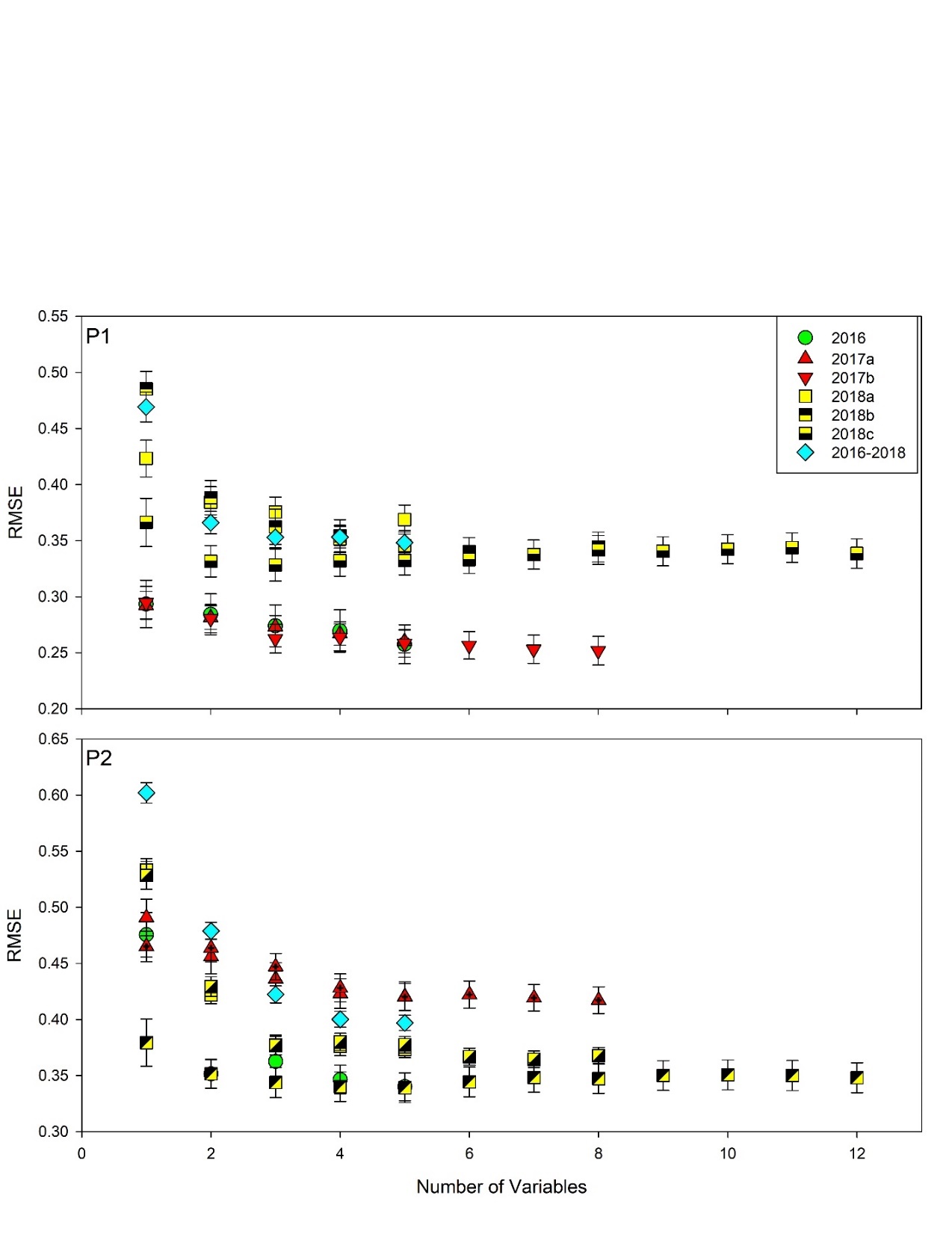


**Supplementary Figure 4**. RMSE reduction for each pond based on the results from the recursive feature elimination exercise.

## Supplementary Tables

| **Supplementary Table 1**. Average values of predictor variables measured in each year. | | | | | | | |  | |  | |  | |  | |  |
| --- | --- | --- | --- | --- | --- | --- | --- | --- | --- | --- | --- | --- | --- | --- | --- | --- |
|  | Pond 1 | | | | | Pond 2 | | | | | |  | |  | |  |
| Variable | 2016 | 2017 | | 2018 | | 2016 | | 2017 | | 2018 | |  | |  | |  |
| EC | 0.90±0.05 | | 1.67±0.03 | | 1.12±0.05 | | 1.03±0.04 | | 0.83±0.04 | | 0.81±0.04 | |  | |  | |
| pH | 8.64±0.06 | | 8.82±0.04 | | 7.65±0.04 | | 7.61±0.10 | | 8.46±0.06 | | 8.17±0.05 | |  | |  | |
| DO | 10.04±0.23 | | 10.39±0.10 | | 10.07±0.12 | | 9.45±0.33 | | 12.97±0.30 | | 14.91±0.32 | |  | |  | |
| SPC | 147.89±1.90 | | 162.51±0.81 | | 148.83±1.75 | | 154.46±1.30 | | 166.95±0.79 | | 142.59±1.13 | |  | |  | |
| C | 26.24±0.11 | | 26.31±0.18 | | 26.99±0.18 | | 26.95±0.23 | | 28.41±0.19 | | 27.54±0.18 | |  | |  | |
| NTU | 7.05±0.27 | | 5.16±0.26 | | 4.20±0.28 | | 36.34±8.33 | | 11.44±1.05 | | 13.86±1.30 | |  | |  | |
| CHL | N.M. | | 3.83±0.41 | | 2.80±0.22 | | N.M. | | 15.30±1.61 | | 19.97±2.02 | |  | |  | |
| PC | N.M. | | 1.15±0.09 | | 0.95±0.11 | | N.M. | | 3.87±0.42 | | 4.86±0.37 | |  | |  | |
| FDOM | N.M. | | 13.04±1.13 | | 20.72±1.09 | | N.M. | | 27.22±0.41 | | 34.90±0.50 | |  | |  | |
| TDN | N.M. | | N.M. | | 6.44±0.11 | | N.M. | | N.M. | | 4.31±0.05 | |  | |  | |
| TDC | N.M. | | N.M. | | 13.96±0.11 | | N.M. | | N.M. | | 15.97±0.08 | |  | |  | |
| NH_4_^+^ | N.M. | | N.M. | | 0.65±0.07 | | N.M. | | N.M. | | 0.23±0.01 | |  | |  | |
| PO_4_^3-^ × 100 | N.M. | | N.M. | | 1.39±0.12 | | N.M. | | N.M. | | 22.82±0.72 | |  | |  | |
| N.M. represents variables that were not measured in a specific year. The ± symbol separates the average from the standard error. EC (log CFU 100 mL^-1^), pH (unitless), DO (mg L^-1^), SPC (µS cm^-1^), C (°C), NTU (NTU), CHL (RFU), PC (RFU), FDOM (µg L^-1^), TDN (mg L^-1^), TDC (mg L^-1^), NH_4_^+^ (mg L^-1^), and PO_4_^3-^ (mg L^-1^). | | | | | | | | | | | | | | | |  |

**Supplemental Table 2**. Probabilities for mean RMSE values to be the same over pairs of years.

|  | Predictor set A | | | Predictor set AB |
| --- | --- | --- | --- | --- |
| Algorithm | 2016-2017 | 2017-2018 | 2016-2018 | 2017-2018 |
|  | Pond P1 | | | |
| SGB | 0.940 | **0.006** | **0.018** | **0.033** |
| kNN | 0.951 | **0.016** | **0.028** | 0.072 |
| MLR | 0.063 | **< 0.001** | 0.255 | **< 0.001** |
| RF | 0.916 | **0.045** | 0.074 | **0.042** |
| SVM | 0.748 | **0.012** | **0.022** | **0.009** |
|  | Pond P2 | | | |
| SGB | **0.044** | 0.236 | 0.509 | 0.209 |
| kNN | 0.438 | 0.813 | 0.811 | 0.534 |
| MLR | 0.351 | 0.432 | **0.008** | 0.188 |
| RF | **0.008** | 0.052 | 0.963 | 0.063 |
| SVM | **0.005** | 0.139 | 0.614 | 0.210 |

**Bold** values indicate statistical significance.

**Supplemental Table 3**. Average mean absolute errors (MAE) of logarithms of *E. coli* concentrations predicted with four machine learning algorithms and multiple linear regression.

|  | Predictor set A | | | | Predictor set AB | | | Predictor set ABC |
| --- | --- | --- | --- | --- | --- | --- | --- | --- |
| ML Algorithm | 2016 | 2017 | 2018 | 2016-2018 | 2017 | 2018 | 2017-2018 | 2018 |
|  | Pond P1 | | | | | | | |
| SGB | 0.193±0.009 | 0.182±0.007 | 0.269±0.010 | 0.247±0.037 | 0.182±0.048 | 0.262±0.008 | 0.236±0.005 | **0.268±0.061** |
| kNN | 0.217±0.012 | 0.193±0.006 | 0.294±0.011 | 0.257±0.044 | 0.193±0.066 | 0.286±0.010 | 0.250±0.006 | 0.281±0.093 |
| MLR | 0.338±0.019 | 0.205±0.007 | 0.435±0.012 | 0.389±0.048 | 0.202±0.047 | 0.408±0.012 | 0.346±0.007 | 0.351±0.076 |
| RF | **0.188±0.010** | 0.182±0.007 | **0.264±0.011** | **0.239±0.042** | **0.175±0.048** | **0.254±0.010** | **0.230±0.006** | 0.269±0.073 |
| SVM | 0.214±0.010 | **0.178±0.008** | 0.288±0.009 | 0.248±0.044 | 0.185±0.045 | 0.288±0.009 | 0.245±0.006 | 0.287±0.072 |
|  | Pond P2 | | | | | | | |
| SGB | 0.265±0.010 | 0.324±0.009 | 0.293±0.006 | 0.305±0.035 | 0.323±0.078 | 0.289±0.006 | 0.310±0.006 | 0.235±0.054 |
| kNN | 0.291±0.011 | 0.318±0.011 | 0.315±0.008 | 0.320±0.041 | **0.322±0.062** | 0.310±0.008 | 0.365±0.008 | 0.306±0.065 |
| MLR | 0.317±0.010 | 0.356±0.009 | 0.347±0.007 | 0.393±0.040 | 0.362±0.062 | 0.334±0.007 | 0.394±0.005 | 0.305±0.059 |
| RF | 0.239±0.008 | **0.319±0.010** | **0.263±0.007** | **0.289±0.029** | 0.323±0.070 | **0.266±0.007** | **0.294±0.005** | **0.226±0.046** |
| SVM | **0.233±0.009** | 0.323±0.009 | 0.275±0.006 | 0.306±0.034 | 0.326±0.064 | 0.288±0.009 | 0.304±0.007 | 0.255±0.050 |

The ± separates the average from the standard error of the mean. The smallest RMSE are shown in bold. Machine learning algorithms: stochastic gradient boosting machines (SGB); k-nearest neighbor (kNN); multiple linear regression (MLR); random forest (RF); support vector machines (SVM). Predictor sets: A – temperature, DO, pH, turbidity, and SPC; AB – all from A and PC, CHL, and *f*DOM ; ABC – all from AB and NH_4_^+^, [PO4]^3-^, TN, and TC.

**Supplemental Table 4**. Average coefficients of determination (R^2^) of logarithms of *E. coli* concentrations predicted with four machine learning algorithms and multiple linear regression.

|  | Predictor set A | | | | | | | | Predictor set AB | | | | | | Predictor set ABC | |  |
| --- | --- | --- | --- | --- | --- | --- | --- | --- | --- | --- | --- | --- | --- | --- | --- | --- | --- |
| ML Algorithm | 2016 | | 2017 | | 2018 | | 2016-2018 | | 2017 | | 2018 | | 2017-2018 | | 2018 | |  |
|  | Pond 1 | | | | | | | | | | | | | | | |  |
| SGB | | **0.720 ± 0.033** | | 0.381 ± 0.011 | | 0.670 ± 0.025 | | 0.628 ± 0.015 | | 0.376 ± 0.032 | | 0.680 ± 0.023 | | **0.677 ± 0.015** | | 0.749 ± 0.021 | |
| kNN | | 0.621 ± 0.016 | | 0.304 ± 0.036 | | 0.607 ± 0.028 | | 0.585 ± 0.019 | | 0.263 ± 0.035 | | 0.611 ± 0.029 | | 0.598 ± 0.022 | | 0.722 ± 0.021 | |
| MLR | | 0.407 ± 0.042 | | 0.244 ± 0.031 | | 0.239 ± 0.023 | | 0.210 ± 0.017 | | 0.221 ± 0.029 | | 0.332 ± 0.022 | | 0.355 ± 0.018 | | 0.587 ± 0.012 | |
| RF | | 0.696 ± 0.033 | | **0.384 ± 0.029** | | **0.687 ± 0.026** | | **0.647 ± 0.018** | | **0.414 ± 0.028** | | **0.697 ± 0.024** | | 0.672 ± 0.019 | | **0.751 ± 0.020** | |
| SVM | | 0.631 ± 0.038 | | 0.364 ± 0.033 | | 0.619 ± 0.027 | | 0.601 ± 0.020 | | 0.325 ± 0.026 | | 0.632 ± 0.024 | | 0.625 ± 0.017 | | 0.670 ± 0.030 | |
|  | | Pond 2 | | | | | | | | | | | | | | | |
| SGB | | 0.614 ± 0.026 | | 0.402 ± 0.029 | | 0.600 ± 0.016 | | 0.515 ± 0.017 | | 0.394 ± 0.030 | | 0.609 ± 0.018 | | 0.511 ± 0.019 | | 0.726 ± 0.017 | |
| kNN | | 0.503 ± 0.035 | | **0.449 ± 0.030** | | 0.562 ± 0.019 | | 0.472 ± 0.018 | | 0.425 ± 0.026 | | 0.552 ± 0.018 | | 0.492 ± 0.021 | | 0.568 ± 0.019 | |
| MLR | | 0.397 ± 0.039 | | 0.295 ± 0.025 | | 0.486 ± 0.019 | | 0.250 ± 0.016 | | 0.279 ± 0.022 | | 0.529 ± 0.020 | | 0.225 ± 0.015 | | 0.592 ± 0.021 | |
| RF | | 0.623 ± 0.035 | | 0.436 ± 0.031 | | **0.669 ± 0.019** | | **0.568 ± 0.015** | | **0.424 ± 0.028** | | **0.667 ± 0.019** | | **0.551 ± 0.016** | | **0.745 ± 0.014** | |
| SVM | | **0.693 ± 0.034** | | 0.411 ± 0.032 | | 0.630 ± 0.017 | | 0.519 ± 0.014 | | 0.384 ± 0.029 | | 0.609 ± 0.020 | | 0.492 ± 0.022 | | 0.675 ± 0.019 | |

The ± separates the average from the standard error of the mean. The smallest RMSE are shown in bold. Machine learning algorithms: stochastic gradient boosting machines (SGB); k-nearest neighbor (kNN); multiple linear regression (MLR); random forest (RF); support vector machines (SVM). Predictor sets: A – temperature, DO, pH, turbidity, and SPC; AB – all from A and PC, CHL, and *f*DOM ; ABC – all from AB and NH_4_^+^, [PO4]^3-^, TN, and TC.

**Supplemental Table 5**. Average normalized root-mean-squared errors (NRMSE) of logarithms of *E. coli* concentrations predicted with four machine learning algorithms and multiple linear regression.

|  | Predictor set A | | | | Predictor set AB | | | Predictor set ABC |
| --- | --- | --- | --- | --- | --- | --- | --- | --- |
| ML Algorithm | 2016 | 2017 | 2018 | 2016-2018 | 2017 | 2018 | 2017-2018 | 2018 |
|  | Pond P1 | | | | | | | |
| SGB | **0.097 ± 0.004** | **0.071 ± 0.003** | 0.126 ± 0.005 | 0.098 ± 0.003 | 0.073 ± 0.003 | 0.124 ± 0.004 | 0.093 ± 0.002 | 0.120 ± 0.004 |
| kNN | 0.110 ± 0.006 | 0.079 ± 0.003 | 0.141 ± 0.005 | 0.105 ± 0.003 | 0.081 ± 0.005 | 0.138 ± 0.006 | 0.102 ± 0.003 | 0.129 ± 0.006 |
| MLR | 0.178 ± 0.013 | 0.082 ± 0.004 | 0.199 ± 0.006 | 0.144 ± 0.003 | 0.082 ± 0.004 | 0.185 ± 0.005 | 0.132 ± 0.002 | 0.160 ± 0.004 |
| RF | 0.100 ± 0.006 | **0.071 ± 0.003** | **0.124 ± 0.005** | **0.095 ± 0.003** | **0.070 ± 0.004** | **0.121 ± 0.005** | **0.092 ± 0.003** | **0.119 ± 0.005** |
| SVM | 0.106 ± 0.005 | 0.073 ± 0.003 | 0.137 ± 0.005 | 0.102 ± 0.003 | 0.074 ± 0.003 | 0.136 ± 0.005 | 0.098 ± 0.003 | 0.133 ± 0.005 |
|  | Pond P2 | | | | | | | |
| SGB | 0.111 ± 0.004 | 0.156 ± 0.005 | 0.166 ± 0.003 | 0.149 ± 0.003 | 0.159 ± 0.006 | 0.163 ± 0.003 | 0.149 ± 0.003 | 0.137 ± 0.004 |
| kNN | 0.123 ± 0.005 | **0.154 ± 0.006** | 0.176 ± 0.003 | 0.157 ± 0.003 | 0.157 ± 0.004 | 0.174 ± 0.004 | 0.151 ± 0.003 | 0.172 ± 0.004 |
| MLR | 0.140 ± 0.005 | 0.171 ± 0.004 | 0.189 ± 0.003 | 0.187 ± 0.003 | 0.173 ± 0.004 | 0.182 ± 0.004 | 0.187 ± 0.002 | 0.170 ± 0.004 |
| RF | 0.102 ± 0.004 | **0.154 ± 0.005** | **0.150 ± 0.004** | **0.141 ± 0.003** | **0.155 ± 0.005** | **0.149 ± 0.003** | **0.143 ± 0.003** | **0.132 ± 0.003** |
| SVM | **0.096 ± 0.004** | 0.157 ± 0.005 | 0.159 ± 0.003 | 0.150 ± 0.003 | 0.160 ± 0.005 | 0.164 ± 0.005 | 0.150 ± 0.003 | 0.148 ± 0.004 |

The ± separates the average from the standard error of the mean. The smallest RMSE are shown in bold. Machine learning algorithms: stochastic gradient boosting machines (SGB); k-nearest neighbor (kNN); multiple linear regression (MLR); random forest (RF); support vector machines (SVM). Predictor sets: A – temperature, DO, pH, turbidity, and SPC; AB – all from A and PC, CHL, and *f*DOM ; ABC – all from AB and NH_4_^+^, [PO4]^3-^, TN, and TC.

**Supplemental Table 6**. Average normalized mean absolute errors (NMAE) of logarithms of *E. coli* concentrations predicted with four machine learning algorithms and multiple linear regression.

|  | Predictor set A | | | | Predictor set AB | | | Predictor set ABC |
| --- | --- | --- | --- | --- | --- | --- | --- | --- |
| ML Algorithm | 2016 | 2017 | 2018 | 2016-2018 | 2017 | 2018 | 2017-2018 | 2018 |
|  | Pond P1 | | | | | | | |
| SGB | 0.076 ± 0.004 | 0.052 ± 0.002 | 0.096 ± 0.004 | 0.071 ± 0.011 | 0.052 ± 0.014 | 0.094 ± 0.003 | 0.067 ± 0.001 | **0.096 ± 0.022** |
| kNN | 0.085 ± 0.005 | 0.055 ± 0.002 | 0.105 ± 0.004 | 0.073 ± 0.013 | 0.055 ± 0.019 | 0.102 ± 0.004 | 0.071 ± 0.002 | 0.100 ± 0.033 |
| MLR | 0.133 ± 0.007 | 0.059 ± 0.002 | 0.155 ± 0.004 | 0.111 ± 0.014 | 0.058 ± 0.013 | 0.146 ± 0.004 | 0.099 ± 0.002 | 0.125 ± 0.027 |
| RF | **0.074 ± 0.004** | 0.052 ± 0.002 | **0.094 ± 0.004** | **0.068 ± 0.012** | **0.050 ± 0.014** | **0.091 ± 0.004** | **0.066 ± 0.002** | 0.096 ± 0.026 |
| SVM | 0.084 ± 0.004 | **0.051 ± 0.002** | 0.103 ± 0.003 | 0.071 ± 0.013 | 0.053 ± 0.013 | 0.103 ± 0.003 | 0.070 ± 0.002 | 0.103 ± 0.026 |
|  | Pond P2 | | | | | | | |
| SGB | 0.088 ± 0.003 | 0.120 ± 0.003 | 0.127 ± 0.003 | 0.113 ± 0.013 | 0.120 ± 0.029 | 0.126 ± 0.003 | 0.115 ± 0.002 | 0.102 ± 0.023 |
| kNN | 0.097 ± 0.004 | 0.118 ± 0.004 | 0.137 ± 0.003 | 0.119 ± 0.015 | **0.119 ± 0.023** | 0.135 ± 0.003 | 0.135 ± 0.003 | 0.133 ± 0.028 |
| MLR | 0.106 ± 0.003 | 0.132 ± 0.003 | 0.151 ± 0.003 | 0.146 ± 0.015 | 0.134 ± 0.023 | 0.145 ± 0.003 | 0.146 ± 0.002 | 0.133 ± 0.026 |
| RF | 0.080 ± 0.003 | **0.118 ± 0.004** | **0.114 ± 0.003** | **0.107 ± 0.011** | 0.120 ± 0.026 | **0.116 ± 0.003** | **0.109 ± 0.002** | **0.098 ± 0.020** |
| SVM | **0.078 ± 0.003** | 0.120 ± 0.003 | 0.120 ± 0.003 | 0.113 ± 0.013 | 0.121 ± 0.024 | 0.125 ± 0.004 | 0.113 ± 0.003 | 0.111 ± 0.022 |

The ± separates the average from the standard error of the mean. The smallest RMSE are shown in bold. Machine learning algorithms: stochastic gradient boosting machines (SGB); k-nearest neighbor (kNN); multiple linear regression (MLR); random forest (RF); support vector machines (SVM). Predictor sets: A – temperature, DO, pH, turbidity, and SPC; AB – all from A and PC, CHL, and *f*DOM ; ABC – all from AB and NH_4_^+^, [PO4]^3-^, TN, and TC.

**Supplemental Table 7.** Average root-mean-squared errors (RMSE), coefficients of determination (R^2^), and mean absolute errors (MAE) of logarithms of *E. coli* concentrations predicted with four machine learning algorithms and multiple linear regression for the combined datasets from each pond over the observation period using predictor set A.

| RMSE | | | | |
| --- | --- | --- | --- | --- |
| ML Algorithm | 2016 | 2017 | 2018 | 2016-2018 |
| SGB | 0.318 ± 0.012 | 0.397 ± 0.009 | 0.382 ± 0.008 | 0.394 ± 0.005 |
| kNN | 0.354 ± 0.011 | 0.400 ± 0.012 | 0.407 ± 0.009 | 0.418 ± 0.007 |
| MLR | 0.441 ± 0.012 | 0.505 ± 0.010 | 0.529 ± 0.009 | 0.543 ± 0.006 |
| RF | 0.307 ± 0.009 | **0.383 ± 0.012** | **0.356 ± 0.008** | **0.377 ± 0.006** |
| SVM | **0.295 ± 0.011** | 0.394 ± 0.010 | 0.383 ± 0.007 | 0.398 ± 0.005 |
| R^2^ | | | | |
| ML Algorithm | 2016 | 2017 | 2018 | 2016-2018 |
| SGB | 0.584 ± 0.029 | 0.608 ± 0.016 | 0.626 ± 0.013 | 0.591 ± 0.010 |
| kNN | 0.509 ± 0.029 | 0.596 ± 0.020 | 0.581 ± 0.016 | 0.546 ± 0.012 |
| MLR | 0.265 ± 0.028 | 0.357 ± 0.018 | 0.302 ± 0.023 | 0.233 ± 0.014 |
| RF | 0.629 ± 0.022 | **0.636 ± 0.022** | **0.677 ± 0.015** | **0.630 ± 0.010** |
| SVM | **0.655 ± 0.023** | 0.610 ± 0.019 | 0.626 ± 0.013 | 0.586 ± 0.012 |
| MAE | | | | |
| ML Algorithm | 2016 | 2017 | 2018 | 2016-2018 |
| SGB | 0.245 ± 0.008 | 0.296 ± 0.007 | 0.295 ± 0.006 | 0.293 ± 0.003 |
| kNN | 0.271 ± 0.008 | 0.288 ± 0.008 | 0.303 ± 0.006 | 0.306 ± 0.004 |
| MLR | 0.339 ± 0.009 | 0.391 ± 0.008 | 0.427 ± 0.007 | 0.424 ± 0.005 |
| RF | 0.229 ± 0.006 | **0.278 ± 0.008** | **0.268 ± 0.005** | **0.278 ± 0.004** |
| SVM | **0.225 ± 0.008** | 0.284 ± 0.007 | 0.288 ± 0.005 | 0.293 ± 0.004 |

Values highlighted in **bold** show the model with the best performance.

**Supplemental Table 8.** Average root-mean-squared errors (RMSE), coefficients of determination (R^2^), and mean absolute errors (MAE) of logarithms of *E. coli* concentrations predicted with four machine learning algorithms and multiple linear regression for the combined datasets from each pond over the observation period using predictor set A and including ‘site’ (e.g. Pond 1 or Pond 2) as a categorical variable.

| RMSE | | | | |
| --- | --- | --- | --- | --- |
| ML Algorithm | 2016 | 2017 | 2018 | 2016-2018 |
| SGB | 0.316 ± 0.013 | 0.365 ± 0.010 | 0.376 ± 0.007 | 0.386 ± 0.006 |
| kNN | 0.354 ± 0.012 | 0.394 ± 0.010 | 0.403 ± 0.010 | 0.393 ± 0.007 |
| MLR | 0.467 ± 0.029 | 0.405 ± 0.008 | 0.526 ± 0.008 | 0.530 ± 0.007 |
| RF | 0.301 ± 0.011 | **0.359 ± 0.009** | **0.352 ± 0.008** | **0.365 ± 0.005** |
| SVM | **0.295 ± 0.009** | 0.369 ± 0.009 | 0.378 ± 0.008 | 0.384 ± 0.006 |
| R^2^ | | | | |
| ML Algorithm | 2016 | 2017 | 2018 | 2016-2018 |
| SGB | 0.604 ± 0.026 | 0.667 ± 0.016 | 0.639 ± 0.013 | 0.606 ± 0.012 |
| kNN | 0.518 ± 0.028 | 0.618 ± 0.019 | 0.586 ± 0.018 | 0.599 ± 0.012 |
| MLR | 0.262 ± 0.031 | 0.593 ± 0.014 | 0.294 ± 0.019 | 0.265 ± 0.012 |
| RF | **0.640 ± 0.022** | **0.674 ± 0.014** | **0.681 ± 0.014** | **0.651 ± 0.009** |
| SVM | 0.634 ± 0.025 | 0.661 ± 0.014 | 0.636 ± 0.015 | 0.616 ± 0.010 |
| MAE | | | | |
| ML Algorithm | 2016 | 2017 | 2018 | 2016-2018 |
| SGB | 0.245 ± 0.010 | 0.271 ± 0.006 | 0.291 ± 0.004 | 0.287 ± 0.004 |
| kNN | 0.271 ± 0.009 | 0.283 ± 0.007 | 0.302 ± 0.007 | 0.289 ± 0.004 |
| MLR | 0.352 ± 0.014 | 0.301 ± 0.006 | 0.422 ± 0.006 | 0.406 ± 0.004 |
| RF | **0.227 ± 0.007** | **0.263 ± 0.006** | **0.264 ± 0.005** | **0.269 ± 0.003** |
| SVM | **0.227 ± 0.007** | 0.264 ± 0.005 | 0.278 ± 0.006 | 0.281 ± 0.004 |

Values highlighted in **bold** show the model with the best performance.
